# Mercury Scenario in Fish from the Amazon Basin: Exploring the Interplay of Social Groups and Environmental Diversity

**DOI:** 10.3390/toxics13070580

**Published:** 2025-07-10

**Authors:** Thaís de Castro Paiva, Inácio Abreu Pestana, Lorena Nascimento Leite Miranda, Gabriel Oliveira de Carvalho, Wanderley Rodrigues Bastos, Daniele Kasper

**Affiliations:** 1 Laboratório de Limnologia, Instituto de Ciências Biológicas, Ecotoxicologia e Ecologia Aquática, Universidade Federal de Minas Gerais, Belo Horizonte 31270-901, MG, Brazil; thaisdecp@gmail.com (T.d.C.P.); lorileitemiranda@gmail.com (L.N.L.M.); 2 Programa de Pós-Graduação em Geociências (Geoquímica), Departamento de Geoquímica, Instituto de Química, Universidade Federal Fluminense, Niterói 24020-141, RJ, Brazil; inacio_pestana@yahoo.com.br; 3 Programa de Pós-Graduação em Ecologia e Recursos Naturais, Laboratório de Ciências Ambientais, Centro de Biociências e Biotecnologia, Universidade Estadual do Norte Fluminense Darcy Ribeiro, Campos dos Goytacazes 28013-602, RJ, Brazil; 4 Laboratório de Estudos Ambientais Olaf Malm, Instituto de Biofísica Carlos Chagas Filho, Universidade Federal do Rio de Janeiro, Rio de Janeiro 21941-900, RJ, Brazil; gabriell.goc@gmail.com; 5 Núcleo Prof. Rogério Vale de Produção Sustentável—SAGE/COPPE, Universidade Federal Do Rio de Janeiro, Rio de Janeiro 21941-972, RJ, Brazil; 6 Laboratório de Biogeoquímica Ambiental, Universidade Federal de Rondônia, Porto Velho 76815-800, RO, Brazil; bastoswr@unir.br

**Keywords:** methylmercury, systematic review, fish consumption, risk calculation, traditional communities, urban communities

## Abstract

The Amazon faces significant challenges related to mercury contamination, including naturally elevated concentrations and gold mining activities. Due to mercury’s toxicity and the importance of fish as a protein source for local populations, assessing mercury levels in regional fish is crucial. However, there are gaps in knowledge regarding mercury concentrations in many areas of the Amazon basin. This study aims to synthesize the existing literature on mercury concentrations in fish and the exposure of urban and traditional social groups through fish consumption. A systematic review (1990–2022) was conducted for six fish genera (*Cichla* spp., *Hoplias* spp. and *Plagioscion* spp., *Leporinus* spp., *Semaprochilodus* spp., and *Schizodon* spp.) in the Web of Science (Clarivate Analytics) and Scopus (Elsevier) databases. The database consisted of a total of 46 studies and 455 reports. The distribution of studies in the region was not homogeneous. The most studied regions were the Madeira River sub-basin, while the Paru–Jari basin had no studies. Risk deterministic and probabilistic assessments based on Joint FAO/WHO Expert Committee on Food Additives (JECFA, 2007) guidelines showed high risk exposure, especially for traditional communities. Carnivorous fish from lakes and hydroelectric reservoirs, as well as fish from black-water ecosystems, exhibited higher mercury concentrations. In the Amazon region, even if mercury levels in fish muscle do not exceed regulatory limits, the high fish consumption can still elevate health risks for local populations. Monitoring mercury levels across a broader range of fish species, including both carnivorous and non-carnivorous species, especially in communities heavily reliant on fish for their diet, will enable a more accurate risk assessment and provide an opportunity to recommend fish species with lower mercury exposure risk for human consumption. The present study emphasizes the need to protect regions that already exhibit higher levels of mercury—such as lakes, hydroelectric reservoirs, and black-water ecosystems—to ensure food safety and safeguard public health.

## 1. Introduction

The Amazon’s biodiversity both holds utilitarian values and supports critical ecosystem services [1], including being an essential provisioning service for the local population: fish production for human consumption [2]. But also, the Amazon has intrinsic value, encompassing its biodiversity and the rights of the human population, especially indigenous and traditional communities, of their existence within this environment [2]. Currently, the total population of Brazil’s North Region is approximately 17,354,884 inhabitants [3], and the vast expanse of the Amazon region is home to a great diversity of cultures and customs. Dietary habits in these communities reflect both their isolation from large urban centers and the relatively better preservation of their environments, owing to the presence of protected areas [4].

According to IBGE (2010, [5]), the average fish consumption in North Brazil, which encompasses a significant portion of the Amazon biome, is around 67 g per capita per day (approximately 24.46 kg per person per year). However, studies in communities along the Lower Amazon, Trombetas, and Purus Rivers [4], as well as the Madeira River [6], report much higher consumption rates of 169 kg and 148.2 kg per person per year, respectively. Moreover, a recent review study reported that fish consumption among urban social groups and traditional social groups (quilombolas, indigenous peoples, and riverine communities) is approximately 149 ± 230 g/person/day and 805 ± 1205 g/person/day, respectively [7]. Consequently, the yearly rate of fish consumption in the Amazon is one of the highest in the world. Fish consumption is vital for human health, providing essential nutrients such as omega-3 fatty acids, proteins, and micronutrients that contribute to overall well-being [8]. Food security is a key focus of the 2030 Agenda for Sustainable Development, aiming to end hunger and ensure access to safe and nutritious food for all (United Nations). Human exposure to chemicals through foods, particularly those of animal origin, is a well-established concern in food safety. This issue is linked to various challenges, including regulatory management [9].

Mercury ranks among the top 10 chemicals of major public health concern, according to the World Health Organization [10]. The primary route of human exposure is through methylmercury (MeHg), a highly toxic organic form of mercury that efficiently bioaccumulates and biomagnifies in aquatic food chains. This exposure occurs when people consume contaminated fish and shellfish [10]. Global awareness of mercury contamination in fish began in the 1950s, following the catastrophic Minamata disaster in Japan [11]. Similarly, in the 1960s, Grassy Narrows and Wabaseemong indigenous communities, Canada, were severely affected by mercury pollution in the aquatic ecosystem [12]. The Amazon region, in particular, has become a critical area of concern, where mercury represents significant health risks, primarily through fish consumption [13]. The rich biodiversity of the Amazon’s aquatic systems supports fish populations that are vital to the ecological, cultural, and economic well-being of local communities [14]. However, environmental degradation poses growing threats to both the health and food security of these populations.

Studies suggest that the primary source of mercury contamination in the Amazon basin is geological, as the regional soil is naturally enriched with the element [15,16,17,18]. However, the region faces numerous anthropogenic impacts that enhance mercury concentrations, such as illegal gold mining, which has been a hotspot for mining since the 1980s [19]. Additionally, activities that alter mercury dynamics in aquatic ecosystems and promote MeHg production, like the construction of hydroelectric dams, further increase its bioavailability and risk to the environment [20,21].

Fish mercury concentrations are influenced by a combination of environmental factors, such as water chemistry, and biological variables, including trophic levels, diet composition, and organism size [22]. In the Amazon, the region’s natural environmental diversity and dynamic conditions play a crucial role in shaping mercury levels in fish. For example, Amazonian waters are typically classified into three types—black, white, and clear—based on their distinct limnological characteristics [23]. Studies have shown that black-water systems tend to present higher mercury concentrations than white- or clear-water systems (e.g., [24,25]). The region’s monomodal flood pulse can also influence mercury bioaccumulation in fish [26,27]. These patterns also affect riverine communities that rely heavily on local fish as a primary food source [17,28]. Consequently, mercury concentrations in human hair frequently exceed the World Health Organization’s safety threshold in these populations [6,29,30], making mercury exposure through fish consumption a major public health concern in the region [31].

There is a significant lack of comprehensive knowledge regarding existing mercury concentration data in the Amazon basin. Current studies are often focused on specific regions, leaving substantial gaps in the overall understanding. Moreover, it is crucial to identify vulnerable regions and social groups, considering the diverse environmental and population characteristics of the Amazon region. To achieve this, it is necessary to access the existing knowledge about mercury concentrations in fish across the region, taking into account factors that may influence these levels. This study aims to evaluate the current knowledge of mercury concentration in fish and verify the exposure of populations of the Amazon basin, considering different social groups (traditional and urban communities) through the calculation of deterministic and probabilistic indices. A database was developed compiling published data on mercury concentrations in six fish genera from the region (three carnivores and three non-carnivores). The dataset also includes reported variables that could influence mercury concentrations, such as individual size, water type of the aquatic ecosystem, and sampling location, to understand the concentrations and exposure of the social groups to mercury.

The hypothesis of the present study is that the distribution of studies on mercury concentrations in fish from the Amazon basin is not uniform, but rather concentrated in specific regions. A growing number of publications is anticipated, driven by increased concern over mercury contamination and the expansion of analytical capabilities. Elevated mercury levels are expected to be found, particularly in carnivorous species, as a result of biomagnification, along with higher exposure risks for traditional social groups. Additionally, mercury concentrations in fish are likely to be significantly influenced by fish size and water characteristics. Social groups and regions requiring greater attention due to elevated risks of mercury exposure through fish consumption are intended to be identified. By highlighting these areas, targeted interventions and policies can be developed to protect those at greatest risk and to ensure food safety for vulnerable social groups. An innovative perspective is also brought by this review, through the integration of ecological, toxicological, and social dimensions to support more effective monitoring strategies and public health protection in the Amazon region.

## 2. Materials and Methods

### 2.1. Systematic Review

For the construction of the Fish Mercury in the Amazon Basin Database, a systematic review was carried out following the PRISMA protocol (“Preferred Reporting Items for Systematic reviews and Meta-Analyses”; [32]). Literature searches were conducted in journals indexed in the Web of Science (Clarivate Analytics) and Scopus (Elsevier) databases. In both databases, the search for the terms “Mercury OR Hg OR Methylmercury OR Monomethylmercury” was done by “topic” (the terms can be found in the title, abstract, or keyword). In addition, six genera of fish species native to the region were searched in all fields (recognizing works that had the term anywhere in the text). Among the fish genera, three were carnivorous genera, *Cichla* spp. (tucunaré), *Hoplias* spp. (traíra), and *Plagioscion* spp. (pescada), and three non-carnivorous, *Leporinus* spp. (piau), *Semaprochilodus* spp. (jaraqui), and *Schizodon* spp. (aracu). Table S1, Supplementary Materials, presents the descriptors used in the searches for the six fish surveyed, which include all the writing possibilities of the genus in question. The time interval adopted was from 1 January 1990 to 31 December 2022.

Duplicate articles were excluded from the two databases (Web of Science and Scopus) using EndNote (Clarivate). Subsequently, the studies that fit the objective of this review were selected by their titles and abstracts. Then, the eligibility of the papers was verified by reading the papers in full. Among the reasons that led to the exclusion of works in the eligibility stage were the following: study area outside the Amazon basin (e.g., regions in northern Colombia and Ecuador); studies without original data (reviews or studies that presented values already used in another study to answer a different question); analysis of fish or muscle tissue samples that have undergone research-induced changes (e.g., analysis of fish exposed to mercury or other substances, and pellet analysis of muscle tissue); and studies that did not contain sufficient data for use, coupled with the inability to contact the corresponding author (e.g., absence of mean values, lack of extractable data from figures, or data grouped by fish characteristics in a way that prevented their use in our analysis). In the end, works (three articles, one dissertation, and one thesis) known to the team that contemplated relevant regions of the Amazon basin, but which were not found during the searches, were included in the research. The steps and details of the systematic review process are shown in the flowchart (Figure S1).

### 2.2. Data Extraction

A total of 46 papers were used to assemble the database on mercury concentration in fish species in the Amazon basin. The data present in the studies were extracted and individualized by genus, and when possible, also by species, collection period, and location. In the end, the database consisted of a total of 46 studies and 455 reports.

The following data were extracted to characterize each report: (i) the name of the first author and year of publication, (ii) genus, species, feeding habit (carnivore or non-carnivore), and trophic guild (iii) sample number, (iv) mean and standard deviation of total length (cm), weight (g), mercury concentrations in muscle (µg/g wet weight), (v) equipment used to determine mercury concentrations, (vi) sampling year, (vii) regional descriptions of the study area (country, state, city, type of ecosystem, sub-basins, and type of water of the aquatic ecosystem where fish were collected), and (viii) geographic coordinates of the collection points.

A recent review and meta-analysis conducted in the Amazon did not observe significant differences in mercury levels between fish sampled in different seasons [33]. Due to this and the lack of precise information on the flooding periods during which the samples were collected, the influence of the flooding pulse on Hg concentrations in fish was not assessed in the present study.

### 2.3. Risk Calculation

For the calculation of deterministic and probabilistic indices of mercury exposure through fish consumption in the region, fish consumption data from Miranda (2024, [7]) was used, covering urban (149 ± 230 g/person/day) and traditional social groups of the Amazon region (quilombolas and riverine and indigenous communities; 805 ± 1205 g/person/day). The Brazilian Institute of Geography and Statistics (IBGE) is a federal government agency that produces and analyzes statistical data on Brazil’s population, economy, and geography. Among its datasets, the IBGE provides regional estimates of fish consumption, offering insights into dietary habits and regional variations, which are valuable for public health and market research. Considering this, one deterministic scenario was calculated using fish consumption data for the North Region of Brazil, as reported by IBGE (2010 [5]; 67 g/person/day). Body weight was gathered from the Brazilian Family Budget Survey [5], with a mean and standard deviation of 67.15 ± 13.5 kg for adults (18 years and older). Body weight parameters were calculated considering that it followed a log-normal distribution (following the method proposed by [34]. For index characterization, the calculated estimated weekly intake (EWI) was compared to the MeHg provisional tolerable weekly intake of 1.6 μg kg bw^−1^ week^−1^, following the Joint FAO/WHO Expert Committee on Food Additives [35]. Deterministic evaluations were based on mean values, while in the probabilistic approach, the distribution parameters of the variables (mean and standard deviation) were used. All parameters used in probabilistic evaluations are reported in the Supplementary Materials (Table S2). A One-Dimensional Monte Carlo simulation was performed using the R package mc2d (Version 0.2.1) [36] to assess the probability of exceeding the Hg provisional tolerable weekly intake, considering the variability of individual parameters. Probabilistic simulations were carried out using more than 1.0 × 10^6^ randomized calculations.

### 2.4. Meta-Analysis

The statistical analyses were conducted using the R programming language [37] through RStudio integrated development environment, version 2024.12.1+563 [38]. In all applicable cases, a predetermined type I error of 5% (α = 0.05) was assumed. This significance level was used to determine the statistical significance of the results obtained in the analyses.

The data extracted from the studies (mean, standard deviation, and sample size) were used to generate a dataset with the same statistical properties as the original data (rnorm function, base package, [37]), assuming a normal distribution. By creating this dataset, the inherent uncertainties within each study and among the selected studies were incorporated into the analysis, and linear models were then employed to analyze the data.

An ANOVA (aov function, base package, [37]) was used to identify effects of sample site and type of water on Hg concentrations found in fish, and multiple comparisons were calculated using Tukey’s test (TukeyHSD, base package, [37]), and significant differences were reported using letter-based representations (HSD.test, agricolae package, [39]). Also, a regression was used to evaluate the effect of fish size on Hg concentrations found in fish. Finally, a multiple regression was used to evaluate the influence of sampling location, fish habit, fish size, and water type on Hg concentrations found in fish. A type III sum of squares was carried out to calculate the variance explained by each variable (R^2^ partial) and all of them together (R^2^ multiple).

When necessary, the data were transformed using a maximum likelihood function (boxcox function, MASS package, [40]) to meet the assumptions of the linear models (normality, linearity, and homoscedasticity of the residuals). The choice of transformation aimed to improve the model’s fit to the data and ensure that the assumptions were met. The models were validated using diagnostic plots [41].

### 2.5. Maps

The maps were projected using the SIRGAS 2000 Datum and the UTM multi-zone projection system. Data sources included geographical information from the Instituto Brasileiro de Geografia e Estatística (IBGE) for administrative boundaries and hydrographic river data. Maps were designed using QGIS (version 3.34.1) software.

## 3. Results

### 3.1. Systematic Review

**Figure 1:**
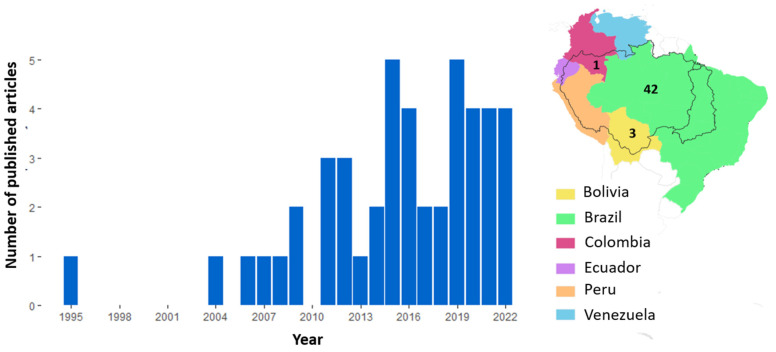
Number of studies published per year (**left**) and number of studies conducted in each country (**right**) with data on mercury concentration in six fish genera (*Cichla* spp., *Hoplias* spp. and *Plagioscion* spp., *Leporinus* spp., *Semaprochilodus* spp., and *Schizodon* spp.) in the Amazon basin. Although the research period spans from 1990 to 2022, no studies were conducted before 1995; therefore, data from this period is not included in the graph.

The 46 studies covered three countries, with 42 studies located in Brazil, and Amazonas was the most studied Brazilian state (Figure 1). Although the research period spans from 1990 to 2022, no studies were conducted before 1995. The first study that investigated mercury concentrations according to the review was [42]. The second publication did not occur until 2004 [43], resulting in a nine-year gap between the first and second studies published. Approximately 85% of the studies were published between 2011 and the present. The years with the highest number of publications were 2015 and 2019 (*n* = 5 each, Figure 1). From the studies published during the research period (1990–2022), the first sampling occurred in 1990 and the last in 2021. Some studies collected samples over years or across different years, and ten studies did not report the year of collection.

Some studies did not specify that the total length was used instead of the standard length. Although length is an important variable for understanding mercury concentrations in fish, some studies did not report such a variable (*n* = 10). Weight was reported in only 63% of the studies.

The most used unit to report mercury concentration was wet weight. Some studies did not report the unit together with the concentrations in the results, and it was necessary to check in the materials and methods if the samples had undergone any drying process before reading for mercury determination, and/or to ask the corresponding author. The detection technique most used to determine mercury concentrations was Cold Vapor Atomic Absorption Spectrometry (*n* = 31, Figure S2).

**Figure 2:**
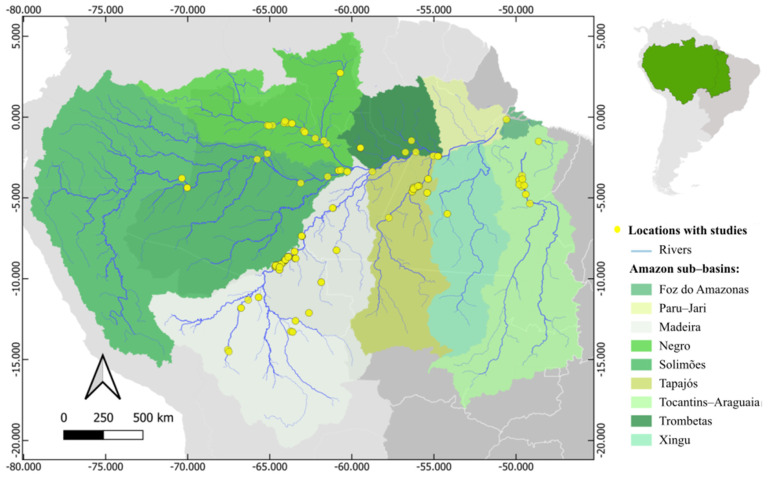
Distribution of studies on mercury concentrations in the six genera (*Cichla* spp., *Hoplias* spp. and *Plagioscion* spp., *Leporinus* spp., *Semaprochilodus* spp., and *Schizodon* spp.) of fish examined in the Amazon basin.

The most studied region was the Madeira basin, while Paru–Jari had no studies (Figure 2). Figures S3 and S4 illustrate the distribution of studies across the Amazon basin according to the fish genera studied. There were more studies that reported collection in natural and hydroelectric ecosystems (*n* = 42) than studies that acquired the specimens in local markets (*n* = 4). Only 12 studies provided data on the limnological variables of the studied area. Most studies determined only mercury concentrations (*n* = 36), while only six studies assessed concentrations of other trace elements, and two studies examined DDT (Dichloro-Diphenyl-Trichloroethane).

Mercury limits in fish muscle for human consumption were assessed in 35 studies. The World Health Organization (WHO) guideline of 0.5 mg/kg wet weight [44] was the most frequently used reference (*n* = 22) to assess the maximum permissible mercury concentration in fish. The second most common reference was Brazilian legislation (*n* = 14), which sets a limit of 0.5 mg/kg wet weight of mercury for non-carnivorous fish and 1.0 mg/kg wet weight of mercury for carnivorous fish [45]. Only two studies applied more conservative recommendations, such as those recommended by the United States Environmental Protection Agency (USEPA), which suggests a maximum safe mercury concentration of 0.3 mg/kg wet weight in fish [46]. Only four studies reported finding no values above the recommended maximum limits. Among the studies that found levels exceeding these limits, predatory/carnivorous species stood out. The proportion of carnivorous specimens that had mercury concentrations above the recommended thresholds varied significantly, from 14% to nearly 100%. Additionally, 11 studies did not report whether the mercury concentrations in fish muscle were considered safe for human consumption based on any recommended limit, and one of these studies focused on mercury intake using the provisional tolerable weekly intake suggested by the Joint FAO/WHO Expert Committee on Food Additives (JECFA) for total mercury (0.23 μg kg^−1^ day^−1^; [47]).

### 3.2. Risk Calculation

**Figure 3:**
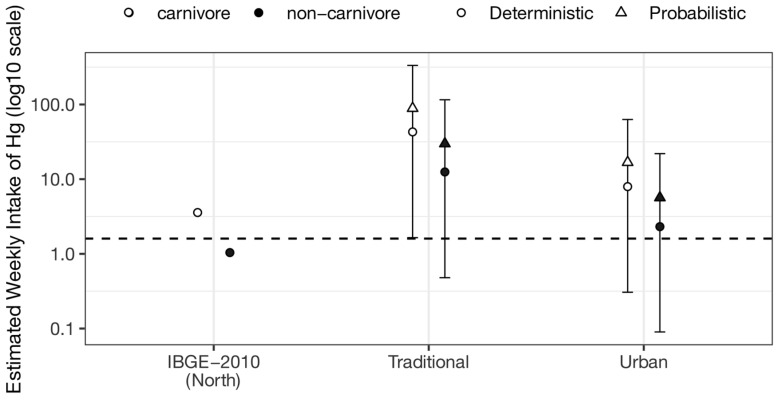
Deterministic and probabilistic indices for mercury exposure through fish consumption, considering fish consumption values for the North Region of Brazil [5] and traditional and urban social groups of the Amazon region [7].

Both the deterministic and probabilistic indices exceeded the MeHg provisional tolerable weekly intake of 1.6 μg kg bw^−1^ week^−1^ recommended by the Joint FAO/WHO Expert Committee on Food Additives [35], except for the index obtained for non-carnivorous fish consumption according to the IBGE ([5]; Figure 3). The probabilistic indices of mercury exposure are presented in Table S3.

### 3.3. Influences on Hg Concentrations

**Figure 4:**
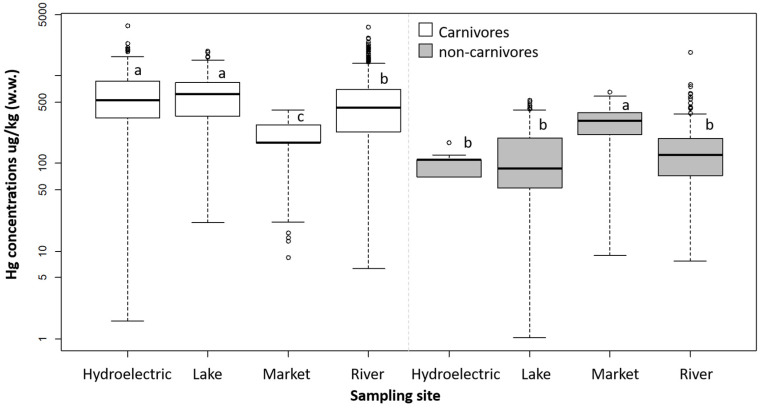
Mercury concentrations in the muscle of fish from the Amazon basin according to their sampling site. Lake: natural lentic aquatic ecosystems. Hydroelectric: lakes and downstream hydroelectric reservoirs. w.w.: wet weight. Feeding habits (carnivorous and non-carnivorous) were analyzed separately. The Hg data were log-transformed to meet the assumptions of the ANOVA. Different letters indicate significant differences according to Tukey’s test, considering a type I error of 5% (α = 0.05).

Based on a multiple regression, the variables sampling location (9.3%, *p* < 0.0001), fish habit (8.1%, *p* < 0.0001), fish size (6.5%, *p* < 0.0001), and water type (4.2%, *p* < 0.0001) together explained 28% (*p* < 0.0001) of the mercury concentrations in the muscle of fish from the Amazon basin. Carnivorous fish exhibited higher mercury concentrations in both hydroelectric reservoirs and natural lakes, while lower concentrations were found in fish purchased at markets (*p* < 0.0001). In contrast, the only significant difference observed for non-carnivorous fish was the elevated mercury levels in individuals acquired from markets (*p* < 0.0006, Figure 4).

**Figure 5:**
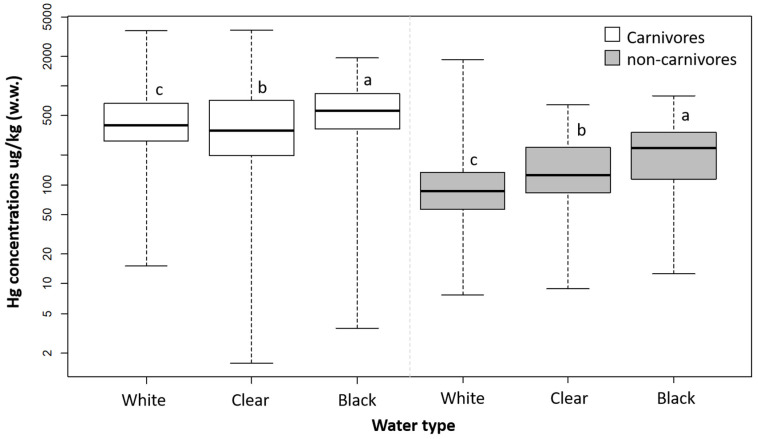
Mercury concentrations in the muscle of fish from the Amazon basin according to the type of water of the aquatic ecosystem [23]. w.w.: wet weight. Feeding habits (carnivorous and non-carnivorous) were analyzed separately. The Hg data were log-transformed to meet the assumptions of ANOVA. Different letters indicate significant differences according to Tukey’s test, considering a type I error of 5% (α = 0.05).

**Figure 6:**
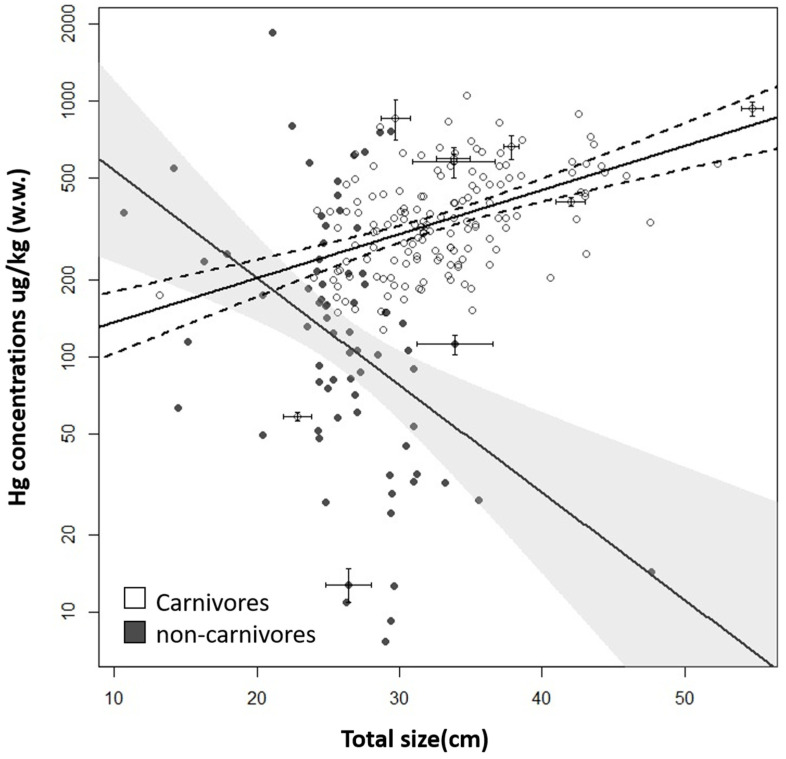
Relationship between mercury concentration in the muscle of fish and their total size in the Amazon basin. X and Y error bars represent the standard deviation, when available. The Hg data were log-transformed to meet the assumptions of regression. Regression statistics are as follows: carnivores = log (Y) = 4.5 + 0.03X; R^2^ = 0.23; *p* < 0.00001; non-carnivores= log (Y) = 7.2 − 0.09X; R^2^ = 0.17; *p* < 0.00001 w.w.: wet weight.

Both carnivorous and non-carnivorous fish exhibited higher mercury concentrations in black-water ecosystems, while the lowest concentrations in carnivorous fish were observed in fish from clear-water ecosystems and in non-carnivores in fish from white-water ecosystems (carnivores: *p* < 0.0001; non-carnivores: *p* < 0.006; Figure 5). Mercury concentrations in fish showed a positive relationship with the size of individuals for carnivorous fish, while the relationship was negative for non-carnivorous fish (Figure 6). Table S4 shows the mean mercury concentration values, standard deviation, and number of samples for the six fish genera extracted from the studies.

## 4. Discussion

### 4.1. Systematic Review

The Madeira River basin stands out among the studied basins. The Amazon region poses significant challenges for sampling, primarily due to its vast and remote areas. Most locations are accessible only by boat or plane, making monitoring studies, especially continuous ones, a complex and expensive undertaking. Additionally, given the enormous size of the Brazilian Amazon basin, covering an area of more than 6.8 million km^2^ [48], there is a limited number of specialized laboratories capable of conducting the necessary analyses to determine mercury concentrations. Some of the institutions with such capabilities include UNIR, UFPA, UFOPA, INPA, UNEMAT, and UFAM. This situation underscores the need for greater investment in infrastructure and accessibility to support environmental monitoring and research in the Amazon. The absence of studies between 1995 and 2004 may be related to limited funding for environmental research in the Amazon during that period, logistical and technical constraints in conducting mercury analyses, and the concentration of research efforts on other topics, fish species, or regions. In addition, some studies may not have been indexed in the selected databases, remaining in local journals, theses, or conference proceedings. Over time, there has been a noticeable increase in the number of studies conducted in the Amazon region, as observed in Figure 1. This growth is likely driven by several factors, including the expansion of research groups with analytical capabilities in the region, improved accessibility to remote areas, and a growing number of researchers dedicated to studying the Amazon. Additionally, rising concerns about human health, increasing public debate, and stricter regulations have heightened the need to understand and monitor mercury contamination. This growing awareness has likely contributed to the observed increase in research efforts over the years.

The six fish genera selected for this study hold significant importance for local consumption [14,49]. Carnivorous species exhibit higher mercury concentrations and have been more extensively studied than non-carnivorous species, likely due to mercury biomagnification and its associated concerns regarding human consumption. However, understanding mercury concentrations in non-carnivorous species is important for both elucidating the dynamics of mercury within the food web and assessing human exposure through fish consumption. In the region, there is a popular saying: “*Quem come jaraqui não sai daqui*” (Those who eat jaraqui—*Semaprochilodus* spp.—don’t leave here), highlighting the cultural value of these fish. However, only four studies assessed the concentrations of mercury in fish of these genera in the study region [47,50,51,52]. This gap in research underscores the need for more comprehensive evaluations of fish safety and mercury levels to ensure the well-being of local communities that rely on these species for their dietary needs.

Although more practical techniques for mercury determination, such as the Direct Mercury Analyzer (DMA), are currently available, Cold Vapor Atomic Absorption Spectrometry (CVAAS), the most traditional technique, was most commonly used method in the studies reviewed from the Amazon basin. CVAAS continues to be favored due to its affordability, reliability, and well-established procedures in environmental monitoring, but also due to practical constraints. In many cases, laboratories are already equipped with CVAAS systems, the personnel are trained in its use, and transitioning to a new technique is neither quick nor easy. Particularly in countries with limited investment in science, migrating to a new technique requires significant funding.

Among the main limitations identified in the reviewed articles are important gaps in the reporting of methodological details, which hinder the interpretation and comparability of the results. The absence of sampling dates in some studies stands out as one of the critical gaps in the data. Moreover, it is possible to highlight other relevant data that are often not reported in the most effective manner, such as specification of the scientific names of the species under analysis, indications of whether total or standard-length measurements of individuals were used, and clarification of whether mercury concentrations were reported in dry or wet weight. Providing these details is crucial for ensuring the accuracy and reproducibility of the research, allowing for proper interpretation and comparison of findings across different studies. A checklist of topics important for studies aiming to understand Hg concentrations in fish is provided in the Supplementary Materials (Table S5).

### 4.2. Risk Calculation

All models, except for the deterministic one based on fish consumption data reported by the [5] and considering only the mercury concentrations of non-carnivorous fish, revealed values exceeding the provisional tolerable weekly intake for MeHg of 1.6 μg kg bw^−1^ week^−1^, as established by the Joint FAO/WHO Expert Committee on Food Additives [35]. The most concerning scenario was observed when considering only carnivorous fish consumption for traditional communities. In this case, almost all simulation values (97.5%) were above the recommended limits (Table S3).

Although alarming, the risks observed in this study align with findings from previous research that assessed mercury exposure risks through fish consumption in the general population of the Amazon [33], specific communities [53,54], and considering characteristics of the social groups under investigation, such as gender and age range depending on the type of fish consumed [55]. While some studies have reported mercury concentrations in fish muscle that do not exceed recommended limits for certain species or in a percentage of the fish analyzed [56,57], the elevated fish consumption rates observed in the Amazonian population, which are often significantly higher than those reported by the IBGE [5] for the North Region, amplify their vulnerability to the exposure risks to any fish contaminant, including mercury. Thus, it is crucial to identify fish species with lower mercury contamination levels and establish their safe weekly consumption rates [33]. These rates vary depending on the type of fish consumed and must be considered when formulating appropriate nutritional guidelines for the local community [55]. Furthermore, the importance of considering the characteristics of aquatic ecosystems, such as ecosystem type and water type, which can influence mercury concentrations as observed in this study, is emphasized (discussed in the next section).

Considering the high risk of mercury exposure due to the elevated fish consumption by local populations, anthropogenic activities that may increase mercury concentrations in the environment and/or alter its dynamics, favoring the formation of MeHg, can further aggravate this risk scenario. Hydroelectric dams, for instance, contribute to the release of mercury from the soil in its toxic form (MeHg) [58]. Consequently, after the construction of a dam, it is common to observe an increase in mercury concentrations in fish [21,59]. In the Amazon region, it has been reported that even 35 years after the construction of a hydroelectric reservoir, mercury concentrations in fish remained elevated [60]. Moreover, in hydroelectric systems constructed in cascade (built consecutively along the same river), mercury concentrations in predatory fish collected upstream increased within the reservoirs along the river [61]. Despite this, the Amazon region remains a target for numerous hydroelectric dam projects [20]. Additionally, the region is threatened by the “arc of deforestation,” which involves extensive biomass burning, deforestation, and mining activities, especially artisanal and small-scale gold mining [62]. These are activities that contribute to increased mercury concentrations in aquatic ecosystems [62,63] and should be better monitored and considered to ensure the food safety of the Amazon region’s population regarding mercury exposure through fish consumption.

**Figure 7:**
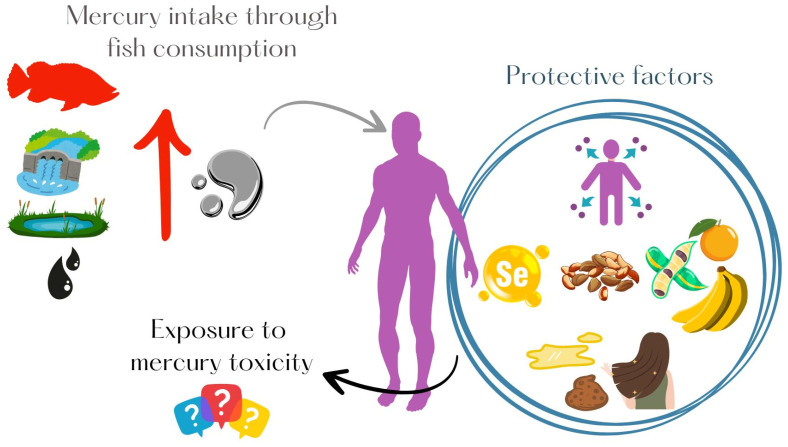
Greater exposure to mercury through fish consumption primarily occurs due to the consumption of carnivorous species, fish from lakes and reservoirs, and fish from black-water ecosystems. Protective factors can mitigate mercury toxicity, including metabolic detoxification processes, the protective role of selenium, a diverse diet rich in protective nutrients (such as Brazil nuts and fruits), and the elimination of mercury through excretory pathways (such as urine, hair, and, mainly, feces). All these factors will determine the toxicity of mercury in the organism.

In the present study, mercury exposure through fish consumption was assessed. However, other factors can also influence mercury toxicity in organisms even after ingestion. Demethylation processes [64], the protective effect of selenium when binding with mercury [65,66], excretion pathways [64,67], and fruit consumption [68] are subjects that can mitigate the toxicity of mercury in the body (Figure 7).

Fish, in addition to its nutritional and cultural importance for populations in the Amazon region, serves as a vital protein source with numerous benefits. However, it is also one of the primary sources of mercury exposure for these populations, often at significant levels. Considering this, actual Brazilian legislation setting limits on mercury concentrations in fish (0.5 mg/kg of mercury for non-predatory and 1.0 mg/kg for predatory fish; ANVISA, 2013) has not effectively protected these populations from mercury exposure through fish consumption, given the high frequency of fish consumption in the region. Furthermore, there is a pressing need for guidance regarding food safety, including the type and origin of consumed fish. Mendes [69], in a study of two riverside communities in the lower Madeira River (São Sebastião do Tapuru and Lago do Puruzinho AM, Brazil), observed that although one community consumed more fish, the mercury concentrations in the hair of its inhabitants were lower than in the other community, which consumed less fish. This difference could be attributed to the higher consumption of non-carnivorous fish species in the former community [69].

With regional development in recent years, communities in previously isolated areas are now transitioning into peri-urban or semi-isolated areas. Consequently, dietary habits among these communities have been changing [70]. While lower mercury concentrations have been observed among inhabitants, especially children—likely due to a shift in fish consumption patterns [69]—these changes have also resulted in statistically significant weight gain and fat accumulation. Additionally, increased blood pressure, higher cholesterol and triglyceride levels, reduced physical activity, and impaired quality of life were noted [70]. Moreover, serum mercury levels remain elevated in a significant portion of the local community and were correlated with body fat percentage [70]. These findings highlight that ensuring food security for populations in the Amazon region, especially regarding mercury exposure, requires comprehensive nutritional guidance and awareness initiatives.

### 4.3. Influences on Hg Concentrations

Carnivorous fish exhibited higher mercury concentrations in lakes and hydroelectric reservoirs, but non-carnivores from these environments had concentrations similar to those from rivers. Mercury methylation is more efficient in lakes than in the main channel, which was reflected in greater bioaccumulation of this element in the biota of the Tapajós [71] and Solimões [72] basins, both in the Brazilian Amazon. In reservoirs, higher mercury concentration values are commonly observed, with the situation being worsened not only by their creation and the lentic nature of these environments but also by the management of these systems [59,60,73]. In the present study, carnivorous fish expressed this difference between habitats in their concentrations. It is possible that non-carnivorous fish did not show this difference because they consume many different food items that may be more or less related to the aquatic system and, therefore, related or not to the bioaccumulation processes that occur in food items originating from the aquatic system. For example, they may feed on plankton or allochthonous material. A similar pattern was observed in the Teles Pires River basin (Brazilian Amazon), where carnivorous fish showed a pattern highly correlated to the environmental dynamics of the aquatic system while non-carnivores were not directly affected [61].

Regarding consumption risk, populations with access to carnivorous fish from markets may be less exposed than those acquiring fish from other sources. However, a larger number of studies would provide a better understanding of this issue. On the other hand, wild non-carnivorous species stand out as a safer option in terms of mercury exposure risk. In the Amazonian context, aquaculture is primarily focused on the cultivation of native fish species [74]. While this activity is still considered to be in a growth phase, data indicate its expansion in the region, particularly in states like Amazonas [75] and Pará [76]. Among the species analyzed in the present study, *Cichla* spp. (tucunaré) stands out as a recommended species for aquaculture [77]. Studies have shown that farmed fish tend to exhibit lower mercury levels compared to their wild counterparts [78,79]. This lower Hg content, observed in previous studies and in the present one for carnivores, can be attributed to differences in diet composition, controlled water quality that could further reduce mercury levels in the fish, and the rapid growth of farmed fish, which results in the dilution of mercury concentrations in their muscle tissue [78,80]. However, since the samples were obtained from markets, it is not possible to determine the origin of the fish. Therefore, mercury concentrations in market fish and their relationship with the fish’s origin still need to be further explored to better understand the patterns of mercury concentrations in market fish and the environmental factors that may influence these concentrations.

Regarding water type, black-water ecosystems stand out, with both carnivorous and non-carnivorous fish exhibiting higher mercury concentrations compared to white and clear waters. As well as lakes and hydroelectric plants, black-water ecosystems exhibit higher mercury concentrations [28]. Black-water ecosystems in the Amazon owe their distinct color and acidity to low concentrations of suspended solids and high levels of dissolved organic acids, which originate from extensive areas of podzol soils in the upstream portion of the basin [24]. Dissolved organic carbon (DOC) forms complexes with mercury, enhancing its transport and accumulation in aquatic ecosystems [24]. Additionally, the low pH characteristic of these waters promotes mercury methylation and its subsequent bioaccumulation in aquatic food chains [24]. Thus, higher concentrations of total mercury and MeHg in both abiotic and biotic compartments of black-water ecosystems, compared to other water types, have been reported in studies. For instance, Vasconcelos et al. [81] observed higher MeHg concentrations in both water and shrimp from a black-water aquatic ecosystem compared to values found in a clear-water ecosystem. Higher mercury concentrations have also been documented in fish, with higher mercury concentrations being associated with elevated DOC levels and the acidic pH characteristic of black-water ecosystems [25]. Furthermore, the elevated mercury levels in black-water aquatic ecosystems are reflected in higher mercury concentrations in the hair of riverine communities that consume fish from these ecosystems [24].

In the present study, mercury concentrations in carnivorous fish showed a positive linear relationship with size, while non-carnivorous fish exhibited a negative linear correlation. However, the correlations were weak, as shown by the low r^2^ values. Roulet and Roulet and Maury-Brachet [82] studied the correlations between biological parameters of fish (weight and size) and their mercury concentrations in several important species from the Amazon region. Among the findings for the genera analyzed in the present study, they observed that *Cichla ocellaris* and *Hoplias malabaricus* clearly show a linear relationship between mercury concentrations and size or weight, while *Plagioscion squamosissimus* and *Leporinus friderici* exhibit no variation with size or weight. On the other hand, *Schizodon* sp. showed a negative nonlinear accumulation. Fish mercury concentrations are commonly correlated with their size and weight due to mercury bioaccumulation in temperate and boreal regions. However, in the Amazon region, fish can show variations in patterns of Hg bioaccumulation [83], and fish Hg bioaccumulation is species-specific [82,84]. Therefore, using size as a proxy to determine mercury concentrations in fish is likely to face limitations and challenges in the Amazon region due to the complex and species-specific patterns of mercury bioaccumulation. In the present study, the correlations took into account different species together; however, since previous research indicates that such relationships are species-specific, we were unable to identify a universal pattern applicable to all species. For this to be possible, future studies should focus on species with well-defined bioaccumulation dynamics. *Hoplias* spp. and *Serrasalmus* spp. could be a potential target [26,82,84]. The *Serrasalmus rhombeus* was noted as a potential indicator in mercury assessments in the Amazon region [61]. Understanding the dynamics of bioaccumulation in species important for human consumption can serve as an indispensable tool for guiding the consumption of these species in a more informed and sustainable manner.

Natural lake, hydroelectric, and black-water ecosystems, along with the social groups dependent on them, should stand out in terms of the need for attention regarding anthropogenic impacts and food safety. These areas must be prioritized for conservation, with efforts focused on mitigating and preventing activities that could elevate mercury concentrations or disrupt its dynamics, promoting methylation and further exacerbating contamination. The present study highlights the need for specific attention to the social groups dependent on these ecosystems, particularly in terms of food safety management and reducing the consumption of carnivorous fish.

## 5. Conclusions

Although a growing number of publications are driven by increasing concern over mercury contamination and advances in analytical capacity, this review reveals that studies on mercury concentrations in fish from the Amazon basin remain unevenly distributed, with research concentrated in specific regions. Traditional social groups, who consume significantly more fish than national census data suggest for the region, face higher exposure risks due to their elevated intake, especially of carnivorous species, which tend to accumulate more mercury through biomagnification. Although fish size is often cited as a predictor of mercury levels, our analysis found only weak correlations, reinforcing previous findings that this relationship is highly species-specific in the Amazon region.

The understanding of mercury exposure through fish consumption in the Amazon region is scarce but critically important, as both population-specific and environmental factors significantly affect exposure levels. Traditional populations, particularly those with diets primarily consisting of carnivorous fish, are at an elevated risk of mercury exposure. Lakes, hydroelectric reservoirs, and black-water ecosystems emerge as key areas for nutritional guidance and environmental conservation, especially in terms of reducing anthropogenic activities that could worsen mercury contamination.

The Amazon’s ecological diversity offers a pathway to reduce mercury exposure risks through dietary adjustments, such as incorporating foods that aid in mercury detoxification and excretion, favoring fish from specific ecosystems, and carefully selecting species for consumption. These measures are especially vital given the high nutritional and cultural value of fish as a protein source in the region. The species-specific aspects of bioaccumulation in fish consumed by humans remain underexplored and could provide crucial insights for improving food safety guidelines. Additionally, continuous monitoring and integrated studies are essential to safeguard both environmental and public health in this unique region.

## Data Availability

The data obtained during the study are available from the corresponding author (Thaís de Castro Paiva) on request.

## Supplementary Materials

The following supporting information can be downloaded at https://www.mdpi.com/article/10.3390/toxics13070580/s1: Figure S1: Flowchart illustrating the systematic review process for analyzing mercury concentrations in fish species from the Amazon basin. Figure S2: Sample preparation and detection equipment used for determining mercury concentrations in studies. Figure S3: Distribution of studies and mercury concentrations in the carnivore genus of fish species examined in the Amazon basin. Figure S4: Distribution of studies and mercury concentrations in the non-carnivore genus of fish species examined in the Amazon basin. Table S1: Descriptors used in the database search for the six fish species of interest in the Amazon basin. Table S2: Values of the parameters used for risk assessment Monte Carlo simulations. Table S3: Monte Carlo simulation values for estimated weekly intake (EQI) of Hg through fish ingestion (quantiles 2.5 to 97.5 % EWI probabilistic distribution) and percentage of cases with EQI above the provisional tolerable weekly intake (PTWI) of 1.6 μg kg bw^−1^ week^−1^, above which the risk exists. Results grouped by social groups and feeding habits of the fish (carnivore or non-carnivore). Table S4: Mean mercury concentration values, standard deviation (SD), and number of samples (n) for the six fish genera extracted from the studies. Table S5: Checklist table with suggested essential items for studies aimed at understanding mercury (Hg) concentrations in fish within the Amazon basin. Reference [85] is citied in the Supplementary Materials.
