# Supplementary material for: Mercury Scenario in Fish from the Amazon Basin: Exploring the Interplay of Social Groups and Environmental Diversity
Source: Toxics. 2025 Jul 10;13(7):580. doi: 10.3390/toxics13070580 (PMC12298900; doi:10.3390/toxics13070580)
Supplement: Supplementary file 1 [file toxics-13-00580-s001.zip › toxics-3714543-supplementary.pdf]

Supplementary material

Table S1. Descriptors used in the database search for the six fish species of interest in the Amazon Basin.

Feeding habits	Gender	Descriptors used
**Carnivores**	*Cichla*	Cichla OR "C. ocellaris" OR "C. kelberi" OR "C. monoculus" OR "C. temensis" OR "C. vazzoleri"
	Hoplias	Hoplias OR "H. aimara" OR "H. malabaricus" OR "H. macrophthalmus"
	Plagioscion	Plagioscion OR "P. squamosissimus" OR "P. auratus" OR "P. montei" OR "P. casattii" OR "P. surinamensis"
**Non-carnivores**	*Leporinus*	Leporinus OR "L. affinis" OR "L. agassizii" OR "L. bimaculatus" OR "L. cylindriformis" OR "L. despaxi" OR "L. fasciatus" OR "L. acutidens" OR "L. freiderici" OR "L. falcipinnis" OR "L. jamesi" OR "L. klausewitzi" OR "L. moralesi" OR "L. nattereri" OR "L. parae" OR "L. octomaculatus" OR "L.

		pachycheilus" OR "L. pachyurus" OR "L. trifasciatus" OR "L. trimaculatus"
	Semaprochilodus	Semaprochilodus OR "S. brama" OR "S. insignis" OR "S. taeniurus" OR "S. varii"
	Schizodon	Schizodon OR "S. fasciatus" OR "S. vittatus" OR "S. brunneus" OR "S. aripuanense" OR "S. arcus" OR "S. alternos"

Standardization of Extracted Data

- To convert concentrations from dry weight to wet weight, a conversion factor of 5 was applied, indicating that dry weight comprises approximately 20% of fresh weight (Rodriguez et al., 2014). *
- In this study, methylmercury was assumed to represent 100% of total mercury in fish samples, as methylmercury typically constitutes 80-100% of total mercury content (EFSA, 2012).
- The ImageJ program was used when necessary to extract data from images to convert fish's standard length to total length.
- The type of water was defined according to the classification or aquatic ecosystem specified in the article. When this information was not available in the text, the author of the study was contacted to obtain the necessary details.
- When the study lacks certain information (e.g., collection year, individual size, sample size, etc.), we attempt to contact the corresponding author of the study.

- When the data could not be obtained through the author or alternative literature searches, the information remained as "not provided."

* Rodríguez Martín-Doimeadios, R.C.; Berzas Nevado, J.J.; Guzman Bernardo, F.J.; Jimenez Moreno, M.; Arrifano, G.P.F.; Herculano, A.M.; Crespo-López, M.E. Comparative study of mercury speciation in commercial fishes of the Brazilian Amazon. *Environ. Sci. Pollut. Res.* **2014**, *21*, 7466–7479.

Table S2. Values of the parameters used for risk assessment Monte Carlo simulations.

Variable	Subgroup	Parameter	Values	Unit	Distribution	Reference
Body weight [1]		mean ± SD	67.15 ± 13.5	kg	normal	IBGE, 2010 (POF-microdata)
THg concentration in Fishes [2]	carnivore	Pooled mean ± Pooled SD	0.51 ± 0.45	$mg\ kg^{-1}$	normal	This Study
	non-carnivore		0.15 ± 0.17			
Fish consumption	Urban	mean ± SD	149 ± 230	$g\ day^{-1}$	normal	Miranda, 2024
	Traditional		805 ± 1205			

[1] Mean and standard deviation of natural log-transformed body weights as done in Portier (2007) but using data from Brazilian population: POF- microdata 2008-2009 (IBGE, 2010); Simulations were truncated in the inferior limit as 0, to not have negative body weight simulations.
[2] Concentrations were truncated in the inferior limit (lowest possible value as 0). The pooled mean was calculated as the weighted average of the group means using sample sizes as weights. The pooled variance was computed by combining the within-group variances (weighted by their degrees of freedom) with the between-group variability (the squared differences between each group mean and the pooled mean, weighted by sample size), and then dividing by the total degrees of freedom.

Table S3. Monte-Carlo simulation values for estimated weekly intake (EQI) of Hg through fish ingestion (quantiles 2.5 to 97.5 % EWI probabilistic distribution) and percentage of cases with EQI above the provisional tolerable weekly intake (PTWI) of 1.6 μg kg bw^{-1} week^{-1} above which the risk exists. Results grouped by social groups and feeding habits of the fish (carnivore or non-carnivore).

Category	Fish Feeding Habit	EWI 2.5%	EWI 97.5%	% of EWI above PTWI
Urban	carnivore	0,3071	63,1528	89,5
Urban	non-carnivore	0,0892	21,9307	72,6
Traditional	carnivore	1,6407	332,4357	97,5
Traditional	non-carnivore	0,4753	116,0879	93,0

Table S4 Mean mercury concentration values, standard deviation (SD), and number of samples (n) for the six fish genera extracted from the studies.

Genera	Mean	SD	n
Cichla	494	381	1535
Hoplias	486	371	448
Plagioscion	609	419	693
Leporinus	148	96	141
Schizodon	170	175	335
Semaprochilodus	83	35	52

Table S5. Checklist table with suggested essential items for studies aimed at understanding mercury (Hg) concentrations in fish within the Amazon Basin.

Item	Description	Check
1	Full scientific and common names of the species	
2	Sample size (n)	
3	Fish length and identification (total or standard length)	
4	Mercury (Hg) concentration (expressed in dry weight or wet weight). Obs. ensure this is indicated in tables and figures.	
5	Date of collection (include year and period; month preferred, but hydrological period is acceptable).	
6	Table with mean values and standard deviations of concentrations and key parameters, such as fish size (supplementary material)	
7	Primary basin associated with the ecosystem	

8	Water color or key water parameters (e.g. pH and dissolved organic carbon)	
9	Geographical coordinates (exact if possible, or approximate with reference to nearby city)	
10	Presence of anthropogenic modifications in the region that could contribute to mercury increase (Industry, agriculture, mining, hydroelectric plants, urbanized areas)	
11	Impact forecast for the region (e.g., planned construction of a hydroelectric dam)	

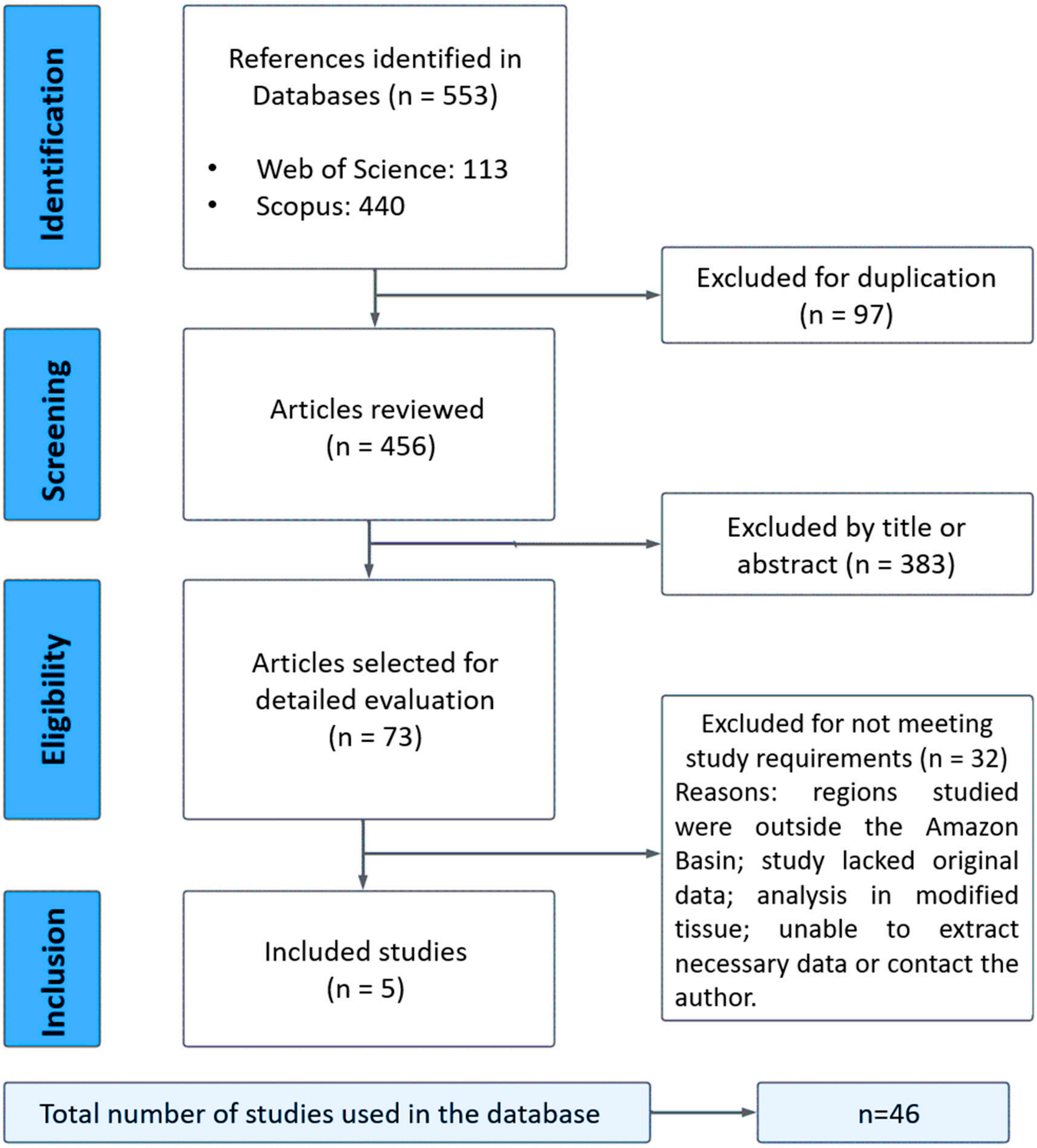

Figure S1. Flowchart illustrating the systematic review process for analyzing mercury concentrations in fish species from the Amazon Basin.

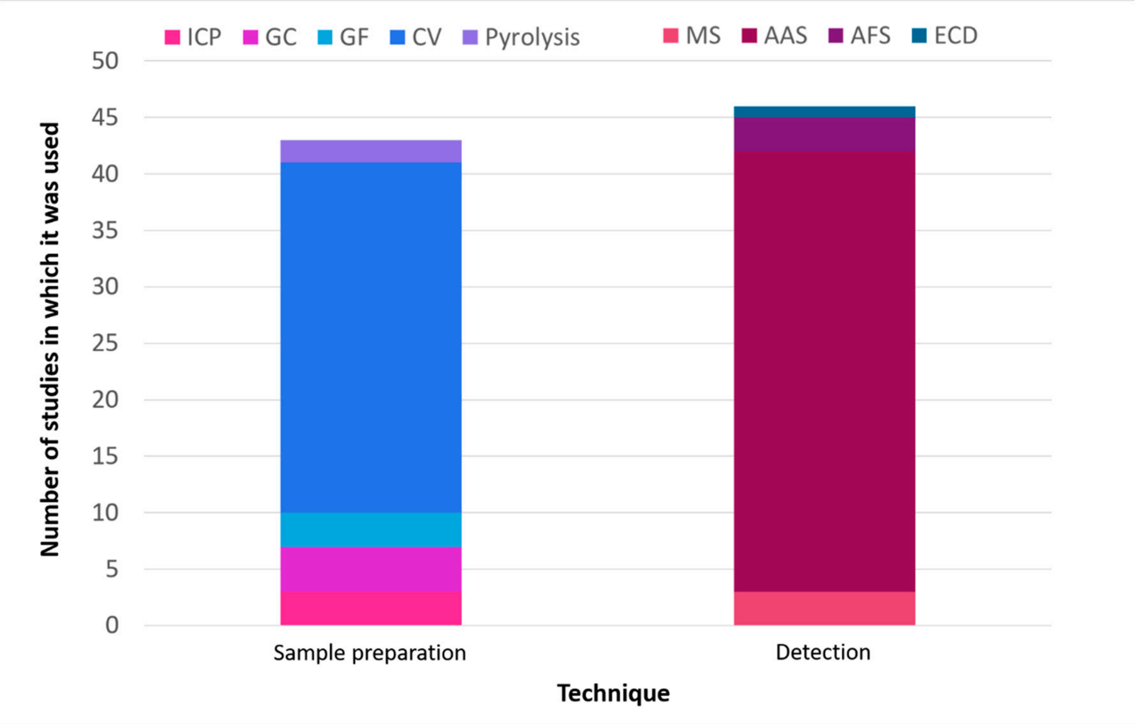

Figure S2. Sample preparation and detection of equipment used for determining mercury concentrations in studies.

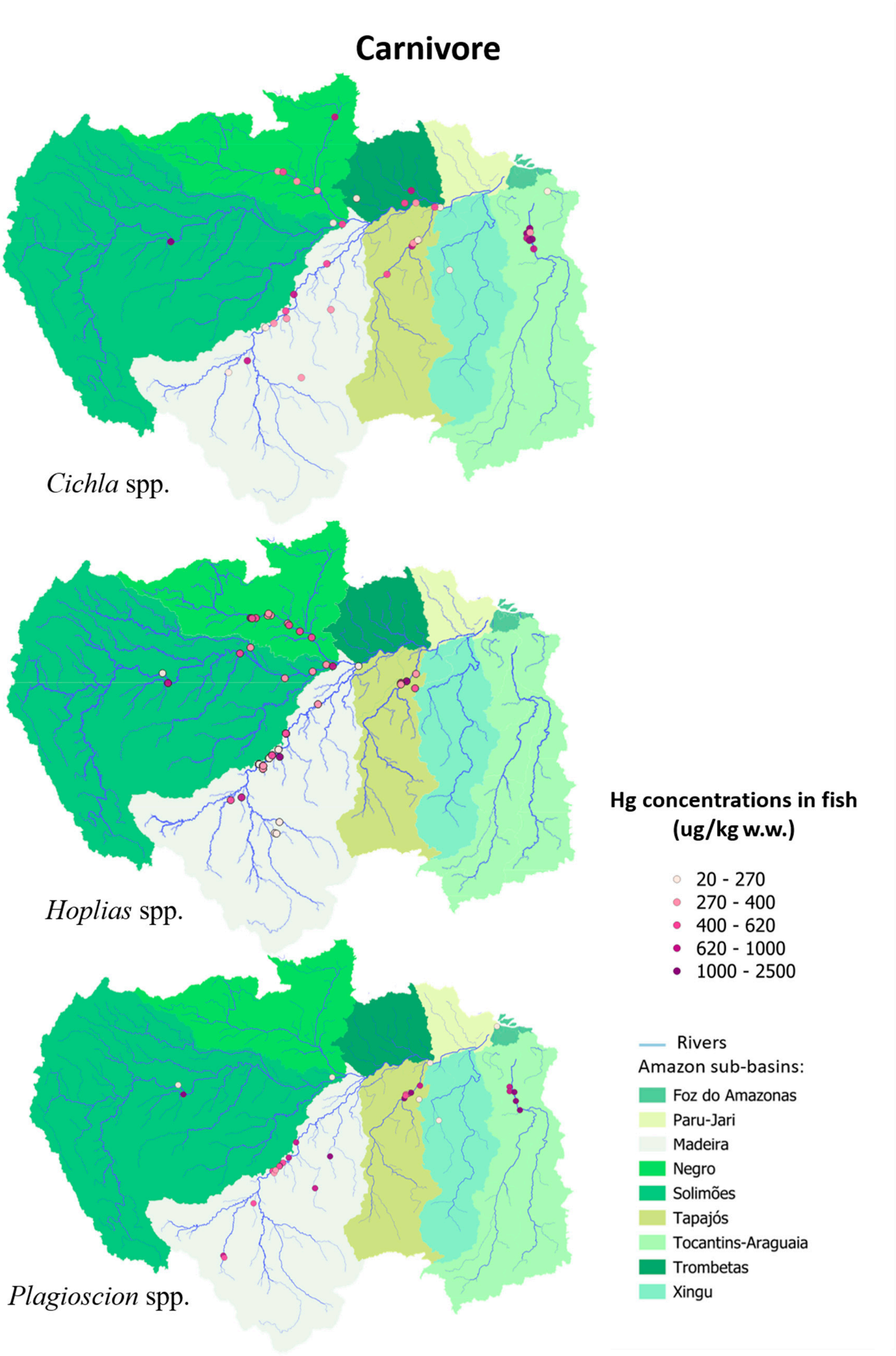

Figure S3. Distribution of studies and mercury concentrations in the carnivore genus of fish species examined in the Amazon Basin.

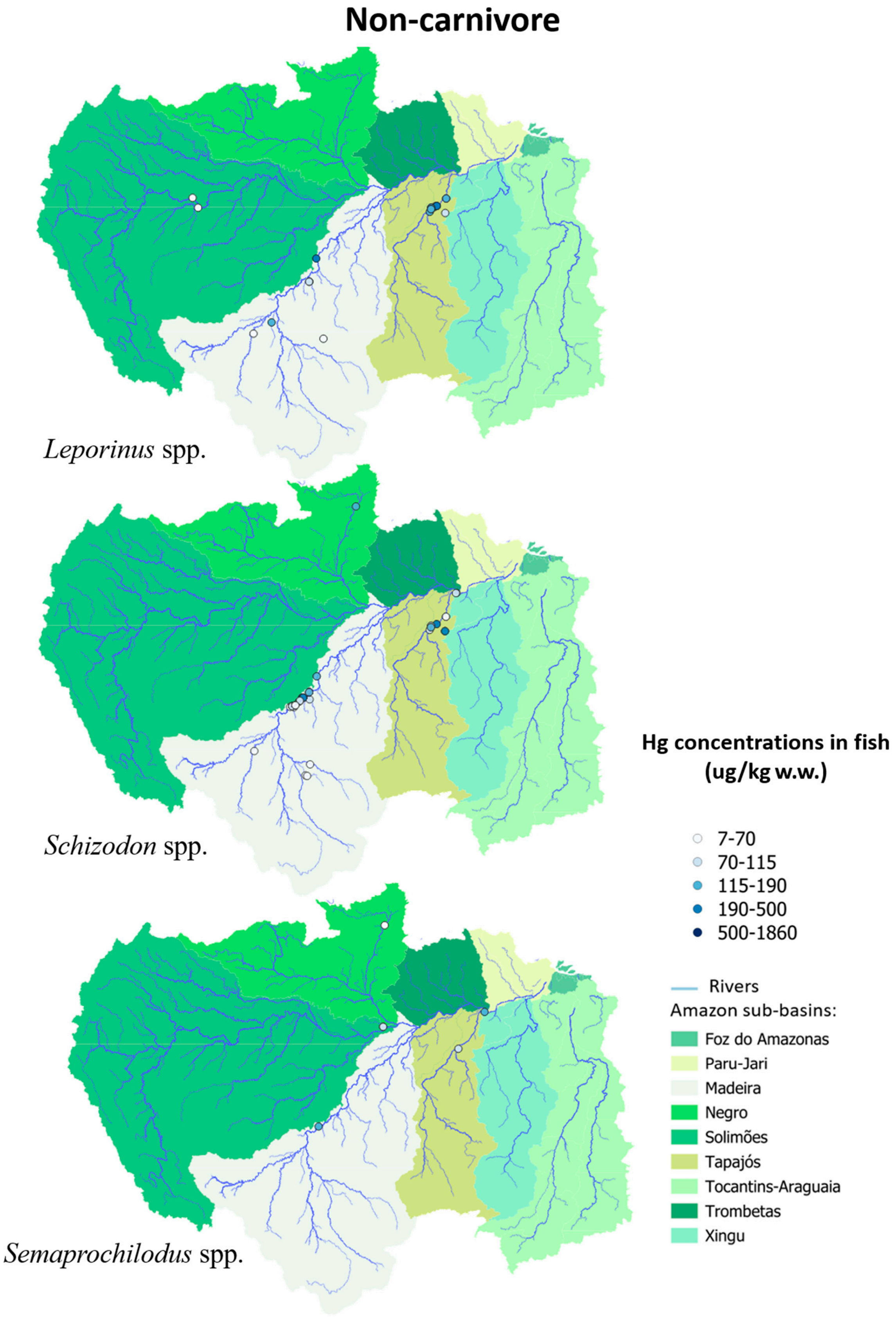

Figure S4. Distribution of studies and mercury concentrations in the non-carnivore genus of fish species examined in the Amazon Basin.
